# Emergence and persistence of ESBL- and carbapenemase-producing *Klebsiella pneumoniae*-related species in Barcelona wastewater treatment plants

**DOI:** 10.1128/spectrum.00621-26

**Published:** 2026-06-30

**Authors:** Victoria Ballén, Karol Delgado, Anna Pinar-Méndez, Carles Vilaró, Belén Galofré, Sara Martí, Aida González-Díaz, Manuel Alcalde-Rico, Sara M. Soto

**Affiliations:** 1 ISGlobal 310844, Barcelona, Spain; 2 Aigües de Barcelona, Empresa Metropolitana de Gestió del Cicle Integral de l’Aigua, Barcelona, Spain; 3 Microbiology Department, Hospital Universitari Bellvitge, IDIBELL-UB 16383, L’Hospitalet de Llobregat, Spain; 4 Research Network for Respiratory Diseases (CIBERES), ISCIII 38176 , Madrid, Spain; 5 CSIC, Universidad de Sevilla, Instituto de Biomedicina de Sevilla, Hospital Universitario Virgen Macarena, Seville, Spain; 6 CIBER Enfermedades Infecciosas (CIBERINFEC), Instituto de Salud Carlos III, Madrid, Spain; 7 Faculty of Medicine and Health Sciences, University of Barcelona (UB), Barcelona, Spain; University of Arkansas for Medical Sciences, Little Rock, Arkansas, USA

**Keywords:** *Klebsiella pneumoniae*, wastewater treatment plants, antimicrobial resistance, heavy metal tolerance genes, virulence, One Health

## Abstract

The World Health Organization classifies extended-spectrum beta-lactamase (ESBL) and carbapenemase-producing *Klebsiella pneumoniae* as critical-priority pathogens due to their high incidence, mortality, transmissibility, rapid resistance acquisition, and limited treatment options. Beyond clinical settings, their detection in wastewater treatment plants (WWTPs) provides an opportunity to assess their prevalence, persistence, and circulation within wastewater systems. This study characterized 37 antibiotic-resistant *K. pneumoniae*-related species strains isolated from two WWTPs in the metropolitan area of Barcelona, analyzing their antimicrobial resistance (AMR) profiles, antimicrobial resistance genes (ARGs), biocide and heavy metal tolerance genes (HMTGs), virulence factor genes (VFGs), biofilm-forming capacity, and conjugation ability. Among them, 70.3% were multidrug-resistant (MDR), and 16.2% were extensively drug-resistant. Whole-genome sequencing revealed diverse ARGs; all strains carried β-lactam resistance genes (14 ESBL and 12 carbapenemase producers), nearly all (96.9%) carried biocide or HMTGs, 64.9% harbored integrases, and all carried VFGs. Core-genome SNP analysis identified closely related strains across sampling periods and treatment stages, suggesting long-term persistence within the wastewater treatment system, despite biological and chemical processes in secondary treatment. Most strains (67.6%) displayed biofilm-forming capacity, and conjugation assays confirmed horizontal gene transfer in five of the seven ESBL-producing strains tested. High-risk clones were predominantly detected in the IFAS secondary treatment stage of the Gavà-Viladecans WWTP. The three strains recovered from the reclaimed water of the Baix Llobregat WWTP were ESBL or carbapenemase producers. Altogether, these results provide genomic and phenotypic evidence of the persistence and circulation of antibiotic-resistant *K. pneumoniae*-related species within wastewater treatment systems.

**IMPORTANCE:**

WWTPs are essential for urban sanitation and environmental protection. Understanding how clinically relevant pathogens, such as ESBL and carbapenemase-producing *K. pneumoniae*-related species strains*,* behave in these settings may inform public health considerations. Investigating the presence and persistence of high-risk MDR pathogens in WWTPs helps identify circulation of AMR, assess the risk of gene transfer, and evaluate the potential for co-selection with other contaminants. This knowledge supports efforts to improve wastewater treatments, strengthen environmental surveillance, and develop integrated One Health strategies to limit the spread of AMR across human, animal, and environmental sectors.

## INTRODUCTION

The World Health Organization (WHO) promotes the One Health framework, recognizing the interconnection of human, animal, and environmental health (1). Disruption of this balance facilitates the emergence and spread of antimicrobial-resistant pathogens, underscoring the need for coordinated, cross-sectoral strategies to address this issue.

Antimicrobial resistance (AMR) is now among the most urgent global health threats, largely driven by inappropriate use of antibiotics and other antimicrobial agents (2). According to the Global Antibiotic Resistance Surveillance Report 2025, one in six laboratory-confirmed bacterial infections in 2023 was caused by antimicrobial-resistant bacteria (ARBs). Between 2018 and 2023, resistance increased in over 40% of monitored pathogen–antibiotic combinations, with average annual increases ranging from 5 to 15%. Among Gram-negative bacteria, *Escherichia coli* and *K. pneumoniae* stand out. Globally, more than 55% of *K. pneumoniae* strains causing bloodstream infections were resistant to third-generation cephalosporins, while resistance to carbapenems and fluoroquinolones continues to rise, narrowing therapeutic options (3).

In Europe, the burden of AMR remains substantial: between 2016 and 2019, the number of resistant infections increased from 685,433 to 865,767, and attributable deaths rose from 30,730 to 38,710, before a modest decline to 801,517 in 2020 (4). Projections indicate that, without effective interventions, AMR could be associated with 8.22 million annual deaths worldwide by 2050, with nearly 2 million of them directly linked to antimicrobial-resistant bacterial infections (5).

Environmental factors exacerbate the AMR crisis. WWTPs play a fundamental role in protecting public health and ensuring water quality. However, they are generally designed to remove organic matter, phosphorus, and nitrogen (6) rather than to eliminate antibiotic residues, heavy metals, or resistant microorganisms (7). As a result, these facilities can inadvertently act as convergence points, where ARBs and antimicrobial resistance genes (ARGs) persist and interact. The persistence of such contaminants within WWTPs can create selective pressures that favor bacterial adaptation and horizontal gene transfer (HGT), which may increase the propagation of ARGs through reclaimed water reuse or effluent discharge into natural ecosystems (8, 9).

Among these pathogens, *K. pneumoniae* is of particular concern in the clinical context as it acts as an opportunistic pathogen capable of causing severe infections. The WHO lists *K. pneumoniae* resistant to carbapenems and/or third-generation cephalosporins as a critical-priority pathogen (10). Its inclusion in the ESKAPE group emphasizes its virulence and rapid acquisition of multidrug resistance (11).

While resistant *K. pneumoniae* strains have been extensively characterized in clinical environments, their prevalence and specific traits in WWTPs require further systematic investigation.

In this study, we addressed this knowledge gap by characterizing 37 antibiotic-resistant *K. pneumoniae*-related species strains obtained from two WWTPs in Barcelona. The research investigated their AMR profiles, ARGs, biocide and heavy metal tolerance genes (HMTGs), virulence factor genes (VFGs), biofilm-forming capacity, and conjugation ability. Through combining phenotypic and genomic analyses, this work provides insights into the persistence, adaptability, and possible dissemination pathways of *K. pneumoniae-*related species in wastewater. These results inform environmental and public health strategies within the One Health framework.

## MATERIALS AND METHODS

### Origin of the *K. pneumoniae*-related species strains

The *K. pneumoniae*-related species strains analyzed in this study were derived from our previous work (12), in which a standardized sampling and processing protocol was applied across the four sampling campaigns to ensure methodological study uniformity. Water samples were collected across different treatment stages of the Baix Llobregat and Gavà-Viladecans WWTPs. During each sampling event, conducted in both winter and summer, 1 L of wastewater was collected for primary and secondary treatment stages of the WWTPs. For tertiary and advanced tertiary effluents, processed sample volumes ranged from 54.6 to 220 L and were concentrated using the Rexeed 25-A (Asahi Kasei Medical Co., Japan) dead-end hollow-fiber ultrafiltration device. Sampling frequency, sampling locations, and collection time (08:00 to 12:00 h) remained constant throughout the study.

As previously described (12), only ARBs were isolated and initially identified using matrix-assisted laser desorption ionization-time of flight mass spectrometry (MALDI-TOF MS) (MALDI Biotyper, Bruker Daltonik GmbH, Germany). Subsequently, ARBs were confirmed by whole-genome sequencing (WGS).

### Antimicrobial susceptibility testing

Antimicrobial susceptibility testing was evaluated using the Kirby-Bauer disk diffusion method for all antibiotics, except for COL, which was tested using the broth microdilution method (BMD). In addition, BMD was also performed to determine the minimum inhibitory concentrations (MICs) of imipenem and meropenem in isolates exhibiting carbapenem resistance by disk diffusion. The antibiotics tested by disk diffusion included amoxicillin-clavulanate (20/10 µg), amikacin (AN, 30 µg), cefepime (FEP, 10 µg), cefotaxime (CTX, 30 µg), ceftazidime (CAZ, 30 µg), chloramphenicol (CHL, 30 µg), ciprofloxacin (CIP, 5 µg), gentamicin (GM, 10 µg), imipenem (IMI, 10 µg), meropenem (MEM, 10 µg), piperacillin-tazobactam (TZP, 10 µg), trimethoprim-sulfamethoxazole (SXT, 1.25/23.75 µg), and tetracycline (TET, 10 µg). Both methods were performed following the Clinical and Laboratory Standards Institute (CLSI 2023) guidelines (13).

According to Magiorakos et al., bacterial strains were classified as resistant to one or two antimicrobial categories, multidrug-resistant (MDR), extensively drug-resistant (XDR), or pandrug-resistant (PDR). MDR strains show resistance to at least one agent in three or more antimicrobial categories, XDR strains show resistance to all but two or fewer categories, and PDR strains exhibit resistance to all tested antimicrobial agents (14).

### Whole-genome sequencing

The DNA of each strain was extracted using the MagMAX DNA Multi-Sample Ultra 2.0 Kit in a KingFisher Flex (Thermo Fisher Scientific, Waltham, USA) and quantified with a Qubit Flex Fluorometer (Thermo Fisher Scientific). Genomic libraries were prepared using the DNA Prep Library Preparation Kit, followed by paired-end sequencing (2 × 150 bp) on a NextSeq platform (Illumina, San Diego, USA). Quality assessment and genome assembly were carried out using the Bactopia pipeline (https://github.com/bactopia/bactopia). WGS data were used to identify ARGs, VFGs, integrase genes, HMTGs, biocide tolerance genes, and MLST. *In silico* MLST typing was performed with the Bactopia MLST module, which also incorporated serotyping results generated using the AMR Finder tool (15). Distances were obtained using the Snippy distance matrix. The phylogenetic tree was midpoint-rooted, and K. *pneumoniae* MGH78578 (CP000647.1) was used as the reference strain. The final tree and its associated metadata were visualized in iTOL (itol.embl.de). The raw sequencing data have been deposited and are publicly available in the ENA under accession codes PRJEB102516 and ERP183908 (title: Resistant *Klebsiella pneumoniae* in Barcelona WWTPs).

### String test

To perform the string test, bacterial colonies were grown on blood agar plates for 20–24 h at 35°C. Using a bacteriology inoculation loop, a single colony was gently touched and slowly lifted vertically. The test was considered positive when a viscous, mucous-like string of ≥5 mm in length formed when the colony was stretched, indicating a hypermucoviscous phenotype commonly associated with hypervirulent *K. pneumoniae* (16).

### Biofilm formation

The biofilm formation protocol was adapted from Ballén et al. (17) with some modifications. Briefly, bacterial strains were cultured on Columbia agar with 5% sheep blood for 18–24 h at 37°C, followed by inoculation into LB broth and incubation for 18–24 h at 37°C with shaking at 300 rpm. Then, bacterial cultures were adjusted to an inoculum of 1 × 10^7^ CFU/mL in fresh LB broth supplemented with 0.5% glucose, inoculated into 96-well polystyrene plates (Nunc Edge 2.0, VWR International), and incubated statically for 48 h at 37°C. After incubation, wells were washed, dried, and stained with 2% (vol/vol) crystal violet for 10 min at room temperature, followed by a final wash and drying. The biofilm-bound stain was solubilized using 33% glacial acetic acid, and biofilm biomass was quantified at 580 nm using a microplate reader (EPOCH 2, BioTek). Strains were classified into biofilm-forming categories based on optical density thresholds, as outlined by Stepanovic et al. (18) as follows: non-biofilm formers (OD ≤ 0.153), weak biofilm formers (0.153 < OD ≤ 0.305), moderate biofilm formers (0.305 < OD ≤ 0.611), and strong biofilm formers (OD > 0.611). Biofilm metabolic activity was evaluated at 24 h and 48 h of biofilm formation using a resazurin reduction assay, adapted from the protocol described by Jiang et al. with some modifications (19). At each designated time point, the supernatant was carefully discarded, and the wells were washed to remove planktonic cells. Subsequently, 0.0015% (wt/vol) resazurin sodium salt solution (75% dye content; Sigma-Aldrich, USA) solution was added to each well. The plates were then incubated at 37°C for 3 h, strictly protected from light to prevent degradation of the photosensitive reagent. Following incubation, relative fluorescence units (RFU) were measured to quantify metabolic activity usinga microplate reader (Infinite M Nano+ 200 PRO, Tecan, Switzerland), and a multimode microplate reader. Non-inoculated culture medium (LB broth supplemented with 0.5% glucose) subjected to the same experimental conditions was used as a negative control to define the background signal and ensure accurate fluorescence readings across both incubation periods. The resazurin assay was included as a complementary approach to assess metabolic activity within developing biofilms and to support the selection of an appropriate incubation time for biofilm quantification.

### Genotype-phenotype association analysis

The association between the phenotypes and genotypes of AMR and biofilm formation was evaluated using Fisher’s exact test. Genes without genotypic variation were excluded due to mathematical infeasibility. For analysis, phenotypes were dichotomized: intermediate and resistant strains were pooled against susceptible ones, and the biofilm phenotype was similarly classified into a binary distribution (biofilm formers and non-biofilm formers). Odds ratios (OR) with 95% confidence intervals were calculated, and because multiple comparisons were made between genes and phenotypes, *P*-values were adjusted using the Benjamini–Hochberg false discovery rate (FDR) procedure to control for type I error inflation, and are reported as adjusted *P*-values (*p_adj_*).

### Conjugation

To evaluate the conjugative ability of the strains under study, seven ESBL-producing strains, resistant to CTX but susceptible to rifampicin (RIF), were selected for analysis. The recipient strain used was *E. coli* CV601, which is resistant to RIF but susceptible to CTX. Potential transconjugants were selected on LB agar plates supplemented with CTX and RIF. The resulting colonies were subsequently analyzed by PCR to confirm the transfer of the resistance genes of interest. Additionally, to ensure that the transconjugants exhibited a banding pattern similar to that of the recipient strain, REP-PCR was done following the protocol established by Vila et al. (20). In addition, the plasmid incompatibility group was identified for each donor strain by PCR (21).

## RESULTS

### Comprehensive identification of isolated bacterial strains

A total of 991 ARBs were isolated from water samples in our previous study (12), of which 37 (3.73%) were identified as *K. pneumoniae* by MALDI-TOF and included in the present study. WGS revealed that these strains belonged to the *K. pneumoniae* complex, including 32 *K. pneumoniae*, four *K. quasipneumoniae* subsp. *quasipneumoniae*, and one *K. variicola*.

Seasonal analysis revealed that eight strains (21.6%) were detected in winter and 29 (78.4%) during summer. The identification of strains across treatment stages indicated that resistant *K. pneumoniae*-related species strains were detected at all treatment stages of the Baix Llobregat WWTP (primary inlet *n* = 2, secondary outlet *n* = 7, tertiary outlet *n* = 2, advanced tertiary outlet *n* = 1). In contrast, at the Gavà-Viladecans WWTP, resistant *K. pneumoniae*-related species strains were detected only at the primary inlet (*n* = 9) and at the integrated fixed-film activated sludge (IFAS) outlet, with the latter showing the highest number of strains (*n* = 16). No *K. pneumoniae*-related species strains were recovered from the reclaimed water of this WWTP.

### Antimicrobial resistance profiles across strains

**Fig 1:**
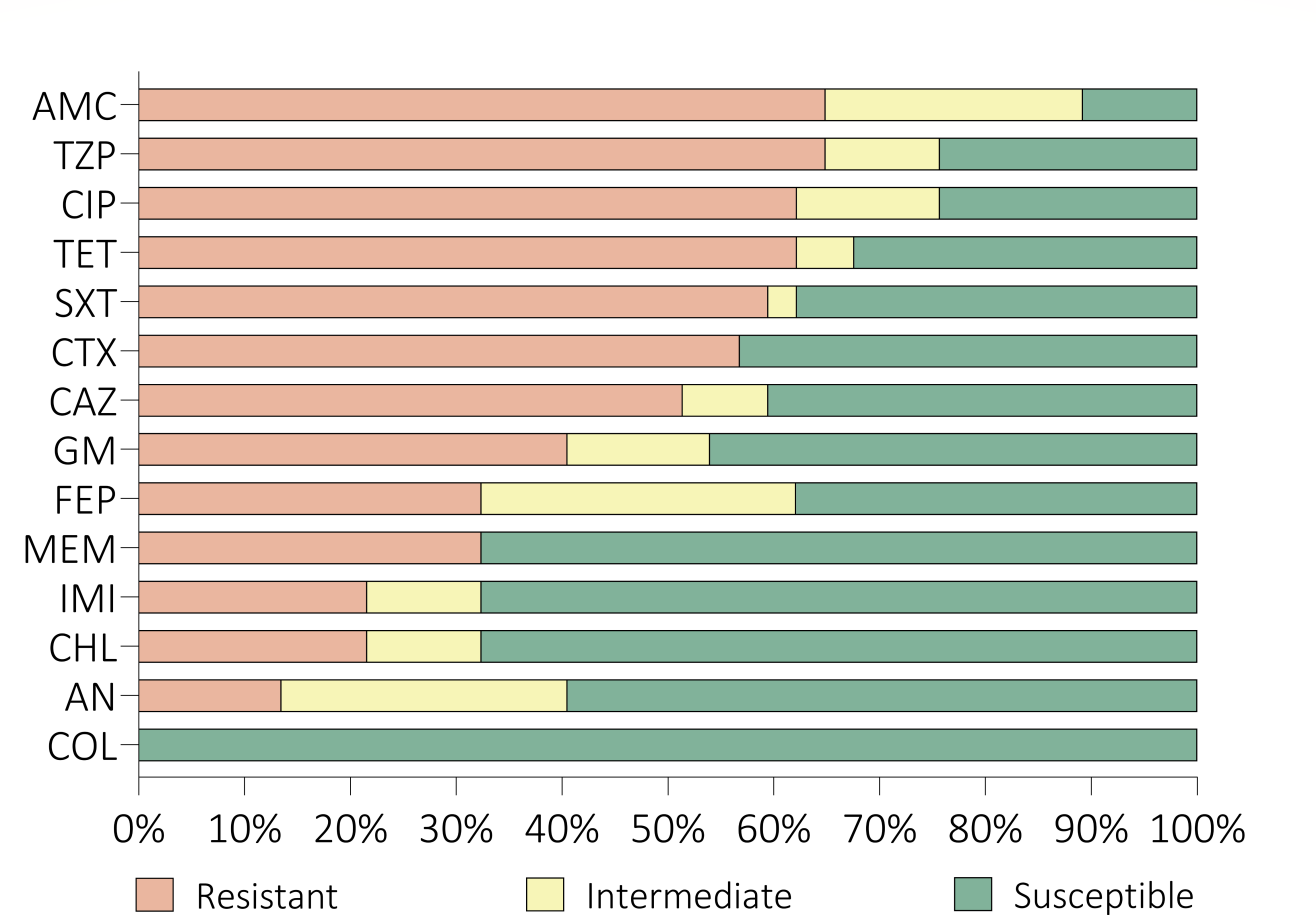
Antimicrobial resistance profiles of K. pneumoniae-related species strains. Each bar represents the percentage of strains classified as resistant, intermediate, or susceptible to each antibiotic tested according CLSI 2023. AMC: amoxicillin-clavulanate, AN: amikacin; CAZ: ceftazidime; CHL: chloramphenicol; CIP: ciprofloxacin; COL: colistin; CTX: cefotaxime; FEP: cefepime; GM: gentamicin; IMI: imipenem; MEM: meropenem; SXT: trimethoprim/sulfamethoxazole; TET: tetracycline; TZP: piperacillin-tazobactam.

As shown in Fig. 1, among the 37 strains belonging to the *K. pneumoniae* species complex, the highest resistance rates are observed for AMC and TZP, each with 24 strains (64.9%). High resistance levels were also noted for TET and CIP, with 23 strains (62.2%) each. More than half of the strains were also resistant to the third-generation cephalosporins, including CTX (*n* = 21, 56.8%) and CAZ (*n* = 19, 51.4%). Resistance to carbapenems was also observed, with 12 strains (32.4%) resistant to MEM and eight to IMI (21.6%). Complete concordance was observed between the broth microdilution MICs and disk diffusion results for all critical carbapenem-resistant isolates. All strains remained susceptible to colistin.

Based on Magiorakos’ antimicrobial resistance classification, five strains (13.5%) displayed resistance to one or two antimicrobial categories, while 26 strains (70.3%) were identified as MDR, and six strains (16.2%) met the criteria for XDR. No PDR strains were detected. At the Baix Llobregat WWTP, the 12 strains were classified as MDR strains. In contrast, at Gavà-Viladecans WWTP, five of the 25 strains (20%) were resistant to one or two antimicrobial categories, 14 strains (56%) were classified as MDR, and six strains (24%) were classified as XDR.

### Genomic insights via whole-genome sequencing

#### Antimicrobial resistance genes

The analysis of 37 *K. pneumoniae*-related species genomes revealed a wide diversity of ARGs, with several strains harboring multiple resistance determinants.

The fosfomycin resistance gene *fosA* was ubiquitous, detected in all strains. Regarding β-lactamase genes, *bla*_SHV_ was the most prevalent gene (*n* = 32, 86.5%), including variants *bla*_SHV-1_, *bla*_SHV-11_, *bla*_SHV-27_, *bla*_SHV-28_, *bla*_SHV-36_, *bla*_SHV-61_, and *bla*_SHV-76_. *bla*_TEM_ genes (*bla*_TEM-1_ and *bla*_TEM-32_) and *bla*_OXA-1_ were each found in 12 strains (32.4%). Within the *bla*_CTX—M_ group*, bla*_CTX-M-15_ was found in 12 strains (32.4%), while *bla*_CTX-M-71_ and *bla*_CTX-M-9_ were each detected in a single strain. Less frequent β-lactam genes included *bla*_OKP-A_ (*n* = 4, 10.8%), *bla*_DHA-1_ (*n* = 2, 5.4%), and single occurrences of *bla*_LEN_ and *bla*_FOX_. Among carbapenemase genes, *bla*_OXA-48_ was the most frequently found (*n* = 7, 18.9%), followed by *bla*_KPC-2_ (*n* = 5, 13.5%), *bla*_VIM-1_ (*n* = 4, 10.8%), and *bla*_KPC-3_ (*n* = 1, 2.7%).

Seven strains (18.9%) co-harbored *bla*_CTX-M-15_, *bla*_TEM_, *bla*_SHV_, and *bla*_OXA-1_, with one also carrying the carbapenemase gene *bla*_OXA-48_.

Among the 12 carbapenemase-producing strains, six carried *bla*_OXA-48_, one *bla*_KPC-2_, and one *bla*_KPC-3_. Three strains co-harbored *bla*_KPC-2_ and *bla*_VIM-1_, while one carried a combination of *bla*_KPC-2_, *bla*_OXA-48_, and *bla*_VIM-1_.

Resistance genes to trimethoprim and sulfonamides were also common: *dfr* genes were found in 20 strains (54.1%), *sul1* in 15 (40.5%), and *sul2* in 13 (35.1%). Four strains carried both *sul1* and *sul2*, and one harbored *sul3* (2.7%).

Tetracycline resistance genes were identified in 21 strains (56.8%), with *tetD* detected in 14 (37.8%) and *tetA* in eight (21.6%). One strain harbored both genes.

Quinolone resistance genes were widespread, with *qnrB* detected in 12 strains (32.4%) and *qnrS* in seven (18.9%). Among these, two strains carried both. Single occurrences of *qnrA* and *qnrE1* were also observed.

Aminoglycoside-modifying enzyme genes were present in 26 strains (70.3%). The most common was *aac(6')-Ib* (*n* = 18, 48.7%), followed by *aph(3')-I* and *aph(6)-Id*, both found in the same 14 strains (37.8%). Additional genes included *aadA* and *aac(3)* (*n* = 10, 27% each) and *rmtf1* (*n* = 4, 10.8%). Less common genes included *ant(2″)-Ia* and *aph(4)-Ia*, each found in one strain (2.7%). Several strains carried multiple aminoglycoside resistance genes, with some harboring up to five.

Among phenicol resistance genes, *catB* was the most prevalent (*n* = 6, 16.2%), followed by *catA* (*n* = 5, 13.5%), while *floR* and *cmIA* were each detected in one strain (2.7%).

Additional ARGs included *arr* (associated with RIF resistance) in four strains (10.8%) and *mph(A)* (macrolide resistance) in six strains (16.2%). The colistin resistance gene *mcr-9.1* was identified in a single KPC-producing strain, isolated from the tertiary outlet (reclaimed water) of the Baix Llobregat WWTP.

### Heavy metal tolerance and biocide tolerance genes

HMTGs were highly prevalent, detected in 29 strains (78.4%). Silver tolerance genes (*sil*) and copper tolerance genes (*pco*) co-occurred in 25 strains (67.6%). Arsenic tolerance genes (*ars*) were present in 20 strains (54%), while mercury tolerance genes (*mer*) were less frequent, occurring in five strains (13.5%). Tellurium tolerance genes (*ter*) were detected in seven strains (19%), and *ncr* (nickel resistance) in one (2.7%).

Additionally, 19 strains (51.4%) simultaneously carried *pco, sil, and ars genes, while two strains harbored the pco, sil, ars,* and *mer* combination, indicating simultaneous tolerance to different heavy metals.

Regarding the association with antibiotic resistance, 19 ciprofloxacin-resistant strains (51%) co-harbored the heavy metal resistance genes *silA* and *pcoD*. A similar co-occurrence was detected among tetracycline-resistant strains, with 10 isolates (27%) carrying both genes, whereas six chloramphenicol-resistant strains (16%) also harbored *silA* and *pcoD*.

Biocide tolerance genes were also common; *qac* (quaternary ammonium compound resistance) were detected in 15 strains (40.5%) and *crcB* (fluoride efflux transporter) genes in two (5.4%).

Remarkably, 22 strains (59.5%) carried 10 or more ARGs, HMTGs, or biocide tolerance genes, with two strains harboring 20 and 21 genes, respectively.

### Virulence factor genes

All strains carried the *wabG* gene, involved in lipopolysaccharide (LPS) biosynthesis. The siderophore gene *entB* (enterobactin) was present in 36 strains (97.3%). Yersiniabactin-associated genes (*ybtS*, *irp2*, and *fyuA*) were found in only 10 strains (27%), whereas *iucA* (aerobactin) and *iroN* (salmochelin) were absent in all strains.

Adhesin-encoding genes showed a high prevalence. *fimD* and *fimH* were found in 36 strains (97.3%), while *mrkD* was identified in 35 strains (94.6%) and *mrkC* in 33 strains (89.2%).

The hypervirulence-associated *rmpA* gene (regulator of the mucoid phenotype) and genes encoding the toxin colibactin were absent from all strains.

### Multilocus sequence typing (MLST)

MLST analysis identified 27 distinct sequence types (STs). The most frequent were ST147 and ST3520 (three strains each). ST15, ST17, ST37, ST193, ST307, and ST1805 were found in two strains each, while the remaining 20 STs were represented by single strains.

Nine high-risk STs, accounting for 15 strains, were identified: ST147 (3), ST15 (2), ST17 (2), ST37 (2), ST307 (2), ST13 (1), ST231 (1), ST268 (1), and ST405 (1). Most high-risk clones (13 out of 15) were detected in the Gavà-Viladecans WWTP, including three at the primary inlet (two ST17 strains from winter and summer samplings, and one ST231 strain from summer) and 10 at the IFAS treatment point (ST13, ST15, ST37, ST147, ST268, and ST307). In contrast, only two high-risk clones were observed in the Baix Llobregat WWTP: ST405 at the primary inlet and ST37 at the secondary outlet.

All high-risk clones were classified as MDR or XDR. Seven produced carbapenemases, nine carried *bla*_CTX-M-15_, and 10 harbored 11 or more different ARGs, together with one to four biocide or HMTGs. These high-risk clones presented relatively low SNP differences among strains, indicating genetic relatedness. Specifically, ST15 showed 40 SNPs, ST147 22 SNPs, and ST307 133 SNPs (Table S1).

**Fig 2:**
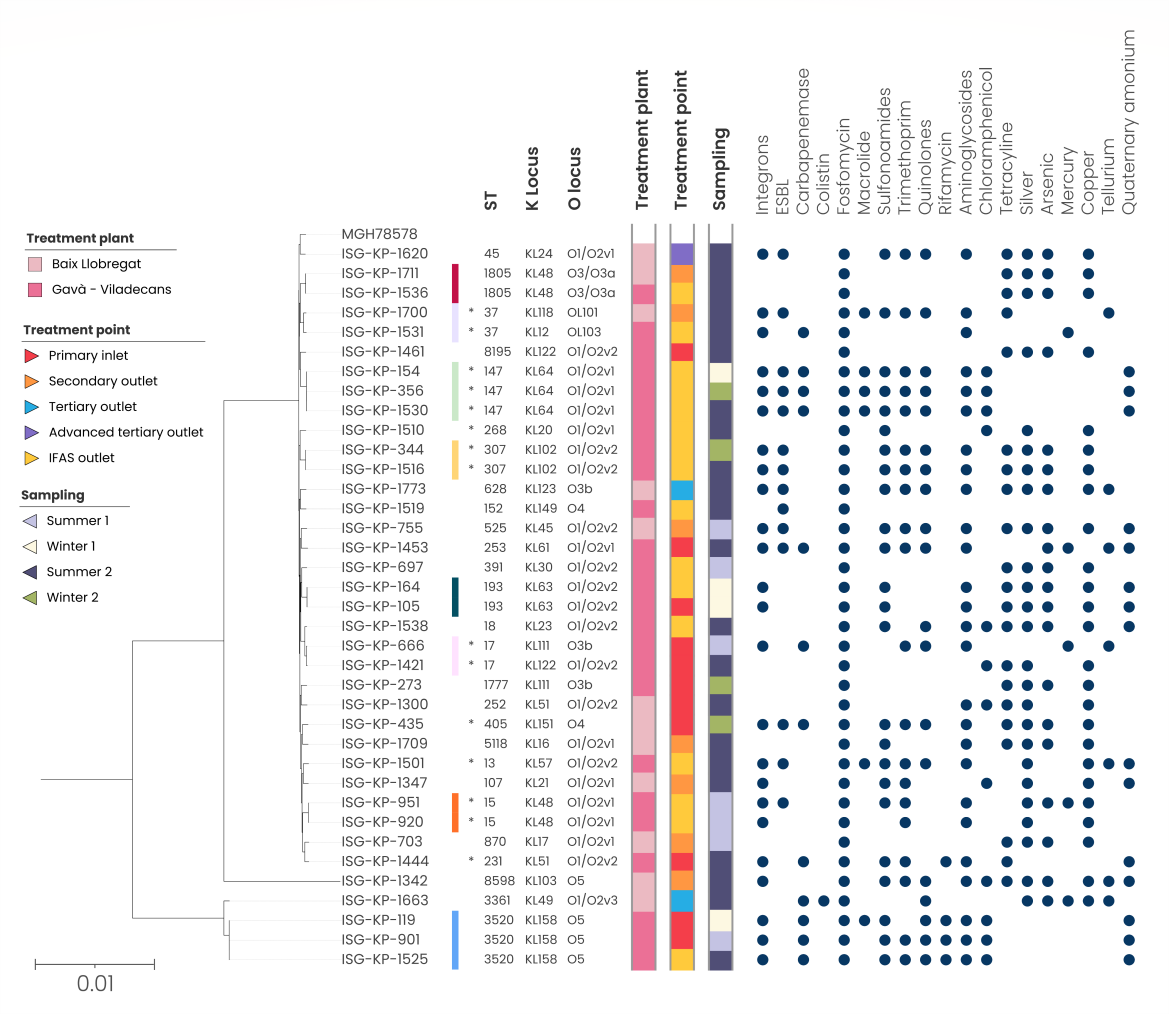
Phylogenetic tree of K. pneumoniae-related species genomes illustrating their multilocus sequence type (ST), K and O loci, integrons, production of extended-spectrum β-lactamases (ESBLs) and carbapenemases, and the distribution of antimicrobial resistance genes across different antibiotic classes, as well as genes conferring tolerance to heavy metals and biocides. Each wastewater treatment plant, treatment stage, and sampling campaign is indicated in separate columns and distinguished by color. Blue dots represent the presence of the corresponding genes. Asterisks (*) denote high-risk clones. Identical colors to the left of the ST column indicate genetically similar strains.

Genomic analysis revealed evidence of recurrent detection of phylogenetically related *K. pneumoniae*-related species clones across different sampling periods and treatment stages in both WWTPs. Three ST147 strains, recovered from the IFAS outlet at the Gavà-Viladecans WWTP during three of the four sampling campaigns, exhibited similar genomic profiles. Similarly, two ST193 strains, obtained during the first winter sampling from both the primary inlet and the IFAS treatment outlet, displayed highly similar genetic profiles, as shown in Fig. 2. Two of the three strains from ST3520 shared the same genotypic profile and ARGs and were detected at the primary inlet (summer sampling 1) and the IFAS treatment stage (summer sampling 2) of the Gavà-Viladecans WWTP, indicating that this clone was detected across separate sampling periods up to the IFAS stage. Two closely related ST1805 strains were identified in both WWTPs during the second summer sampling: one from the secondary outlet of the Baix Llobregat WWTP and the other from the IFAS treatment stage of the Gavà-Viladecans WWTP, demonstrating the presence of the same ST in both facilities. Finally, two ST307 strains with genetically similar profiles were recovered from the IFAS treatment point of the Gavà-Viladecans WWTP during the second winter and summer samplings, suggesting their continued presence at this site.

### Integrons

Class 1 integrase was detected in 23 strains (62.2%), while class 3 integrase was identified in two (5.4%). No class 2 integrase was detected. Seven class 1 integrase-positive strains were found at the Baix Llobregat WWTP, whereas the remaining 16 were isolated from the Gavà-Viladecans WWTP. Both class 3 integrase-positive strains originated from the Gavà-Viladecans WWTP; one of these strains co-harbored *intI1* and *intI3*.

Ten of the 12 carbapenemase-producing strains carried integrases. Among the 24 integrase-positive strains, 21 harbored *sul* genes, and 14 carried *qac* genes. Notably, 14 of the 15 *qac*-positive strains also carried *sul* genes and integrases, emphasizing the possible co-occurrence of antimicrobial and biocide tolerance genes and suggesting integron-mediated co-selection.

The genomic features identified by WGS are summarized in Fig. 2.

### Evaluation of the string test

The hypermucoviscosity phenotype, a key indicator of hypervirulent *K. pneumoniae*, was assessed using the string test. Only one strain, isolated from the IFAS treatment point at the Gavà-Viladecans WWTP, exhibited a positive result, confirming the hypermucoviscous phenotype. However, this strain did not harbor the *rmpA* or *magA* genes typically associated with this characteristic.

### Evaluation of biofilm production

**Fig 3:**
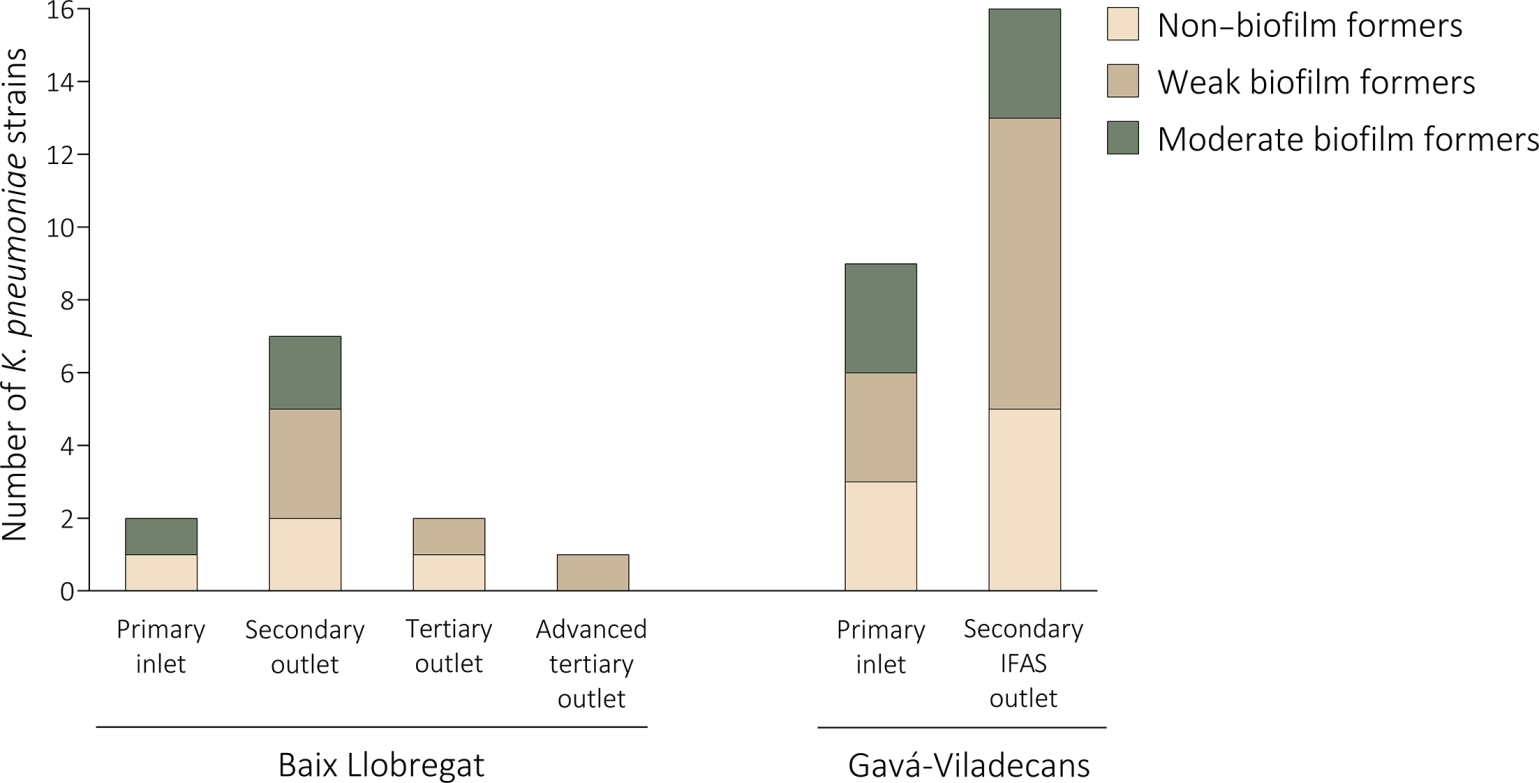
Distribution of K. pneumoniae-related species strains and their biofilm-forming capacity across different treatment stages in the wastewater treatment plants.

Of the 37 *K. pneumoniae*-related species strains, 12 (32.4%) were classified as non-biofilm formers, including eight from the Gavà-Viladecans WWTP and four from the Baix Llobregat WWTP. Sixteen strains (43.2%) showed weak biofilm formation, with 11 identified at the Gavà-Viladecans WWTP and five at the Baix Llobregat WWTP. The remaining nine strains (24.3%) demonstrated moderate biofilm-forming ability, comprising six from the Gavà-Viladecans WWTP and three from the Baix Llobregat WWTP. No strains were identified as strong biofilm formers. Among the three strains recovered from reclaimed water of the Baix Llobregat WWTP, two exhibited weak biofilm formation, while one was classified as a non-biofilm former. Figure 3 illustrates the spatial distribution of *K. pneumoniae*-related species strains across the treatment stages and their biofilm-forming capacity. Analysis by ST revealed that biofilm formation developed under laboratory conditions was variable even among genetically related strains. Both ST37 strains were biofilm formers, whereas only one of the two ST307 strains formed biofilm. Similarly, both ST193 strains were biofilm producers, while within ST17 and ST15, only one of the two strains in each group demonstrated biofilm formation. For ST3520, two of the three strains were capable of forming biofilm.

**Fig 4:**
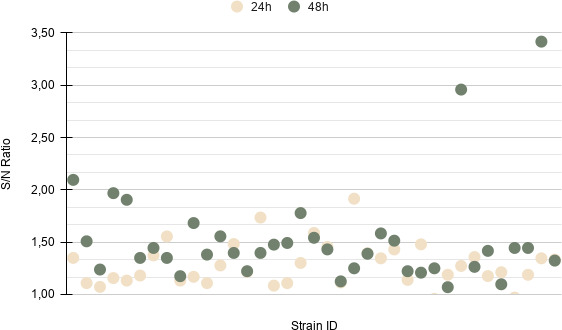
Signal-to-Noise (S/N) Ratio. Comparison of resazurin-based metabolic activity at 24h and 48h of biofilm formation.

The use of a 48-hour incubation period was supported by the resazurin-based metabolic activity assay. At 24 h, fluorescence signals were frequently close to background levels, limiting reliable discrimination between strains. In contrast, 48-hour incubation resulted in improved signal-to-noise separation, enabling more reliable detection of metabolically active biofilms (Fig. 4).

Overall, most strains exhibited stable or increased metabolic activity between 24 h and 48 h, consistent with biofilm maturation.

### Genotype–phenotype association analysis

#### Antibiotic susceptibility and non-susceptibility profiles

The genotype–phenotype associations between resistance genes and antibiotic non-susceptibility profiles are summarized in Table 1. The matrix is structured to contrast strong predictors against borderline trends and non-significant determinants across each antibiotic. For CHL, the genes *catA2*, *cmlA*, and *floR* were excluded from formal statistical analysis due to low prevalence (*n* = 1). Similarly, due to zero phenotypic variation (all 37 isolates were colistin-susceptible) and the presence of *mcr*-9.1 in only a single isolate, formal statistical testing was unfeasible for the colistin group, though the absolute absence of phenotypic resistance within this environmental collection remains an important descriptive observation. Consequently, these specific cases are categorized as excluded or unfeasible within the table’s interpretive framework.

**TABLE 1:** Statistical evaluation of genotype–phenotype associations between antimicrobial resistance genes and non-susceptibility profiles^*a*^

Phenotype	Genotype	OR	p_adj_	Statistical significance	Interpretation
β-lactam resistance					
ESBL phenotype	*bla*_CTX-M-1_	∞	<0.05	HS	[++]
ESBL phenotype	*bla*_TEM,_ *bla*_SHV_	N/A	>0.05	NS	N/A
CTX	*bla*_CTX-M-1_	0	<0.001	HS	[++]
	*bla*_OXA-1_	0.07	<0.05	S	[+]
CAZ	*bla*_CTX-M-1_	0.06	<0.05	S	[+]
FEP	*bla*_CTX-M-1_	0	<0.001	HS	[++]
	*bla*_OXA-1_	0.09	<0.05	S	[+]
AMC or FOX	*bla*_CTX-M-1_	N/A	>0.05	NS	N/A
Carbapenem resistance					
IMI	*bla*_KPC-2_	0	<0.05	S	[+]
	*bla*_OXA-48_	0	<0.001	HS	[++]
	*bla*_VIM-1_	0	<0.05	S	[+]
MEM	*bla*_KPC-2_	0	<0.05	S	[+]
	*bla*_OXA-48_	0	<0.001	HS	[++]
	*bla*_VIM-1_	0	<0.05	S	[+]
Sulfonamide resistance					
SXT	*dfr*	0.02	<0.0001	HS	[++]
	*sul2*	0	<0.001	HS	[++]
	*sul1*	0.14	<0.05	S	[+]
Tetracycline resistance					
TET	*tetD*	0	<0.01	HS	[++]
	*tetA*	0	<0.05	S	[+]
Aminoglycoside resistance					
AN	*aac(6'*)	0.18	0.0569	Borderline NS	N/A
	*rmtF1*	0	0.0569	Borderline NS	
	*aac (3*)	N/A	>0.05	NS	N/A
GM	*aac(6'*)	0.04	<0.0001	HS	[++]
	*aac(3*)	0	<0.01	HS	[++]
GM/AN	*aadA, aph(3’), aph(6*)	0	>0.05	NS	N/A
Quinolone resistance					
CIP	*qnrB*	0	<0.05	S	[+]
	*qnrS*	N/A	>0.05	NS	N/A
Amphenicol resistance					
CHL	*catA1*	0	<0.05	S	[+]
	*catB*	0	<0.05	S	[+]
	*catB3*	0.22	>0.05	NS	N/A

^*a*^ Borderline NS, borderline non-significant trend (0.0500 < *p_adj_* < 0.0600); HS, highly significant (*p_adj_* < 0.01); N/A, not applicable; NS, not significant (*p_adj_* > 0.05); OR, odds ratio; OR = 0 indicate strong association with susceptibility; *p_adj_*, adjusted *P*-value using the Benjamini–Hochberg procedure (Fisher’s exact test); S, significant (*p_adj_* < 0.05); [++], primary genotype–phenotype predictor (highest statistical weight or lowest *P*-value); [+], confirmed predictor or direct non-susceptibility marker.

### Analysis of associations between virulence genotypes and phenotypes

Among the VFGs and the biofilm formation phenotype studied, all five fimbrial genes (*fimD*, *fimH*, *mrkC*, *mrkD*, and *ycfM*) were highly prevalent across all biofilm phenotype categories, with positivity rates consistently above 80% and frequently reaching 100%. Their distribution showed minimal variation between the no biofilm, weak biofilm, and moderate biofilm groups, indicating that the presence of these fimbrial genes is essentially ubiquitous in the collection and does not differentiate between biofilm phenotypes. Due to the near-ubiquitous distribution of some genes (*entB*, *fimD*, and *fimH*), statistical association analyses could not be reliably performed.

Hypermucoviscosity was detected in a single strain and was excluded from the statistical association analysis due to lack of variation.

### Horizontal gene transfer assessed by conjugation experiments

Conjugative transfer of ARGs was successfully demonstrated in five of the seven ESBL-producing *K. pneumoniae*-related species strains tested. A summary of the successful conjugation events is provided in Table 2.

**TABLE 2:** Summary of successful conjugation experiments

Treatment plant	Treatment point	Strain	ST	Harbored genes	Transferred genes	Plasmid incompatibility groups
Baix Llobregat WWTP	Secondary outlet	755	307	*bla*_CTX-M-1*,*_ *bla*_TEM*,*_ *bla*_SHV_	*bla*_CTX-M-1*,*_ *bla*_TEM_	IncFIB
	Tertiary outlet	1773	628	*bla*_CTX-M-1*,*_ *bla*_TEM_	*bla*_CTX-M-1*,*_ *bla*_TEM_	IncFIB
	Adv. Tertiary outlet	1620	45	*bla*_CTX-M-1*,*_ *bla*_SHV_	*bla*_CTX-M-1*,*_ *bla*_SHV_	IncFIB
Gavá-Viladecans WWTP	Secondary IFAS outlet	344	307	*bla*_CTX-M-1*,*_ *bla*_TEM*,*_ *bla*_SHV_	*bla*_CTX-M-1*,*_ *bla*_TEM_	IncFIB
		951	15	*bla*_CTX-M-1*,*_ *bla*_TEM_	*bla*_CTX-M-1*,*_ *bla*_TEM_	IncFIB

## DISCUSSION

Although conventional wastewater treatment effectively removes common water contaminants, ARBs and ARGs are not systematically monitored and may remain in treated effluents (22). Among ARBs, *K. pneumoniae* stands out as a major concern due to its opportunistic pathogenicity, its array of VFGs, and its remarkable capacity to acquire and disseminate ARGs, often through mobile genetic elements (MGEs) that facilitate HGT (23).

More than half of the *K. pneumoniae*-related species strains showed resistance to multiple antibiotic families, including β-lactams, tetracyclines, fluoroquinolones, and sulfonamides. Seventy percent were classified as MDR. All strains carried β-lactam resistance genes, predominantly *bla*_SHV_. This distribution contrasts with studies from other regions, such as Poland, where *bla*_GES_ was more prevalent (24).

Several high-risk clones were identified, emphasizing the global dissemination of epidemic *K. pneumoniae* lineages that combine VFGs with ARGs (25–31). The detection of these clones in wastewater may provide valuable information for public health.

The most frequently identified STs—ST15, ST147, and ST307—are globally distributed high-risk lineages commonly associated with ESBLs and carbapenemase production, as well as severe healthcare-associated infections.

ST15 has been linked to hybrid virulence-resistant plasmids and the production of ESBLs and carbapenemases (32). ST147 is frequently associated with *bla*_NDM_, *bla*_OXA-48_, *bla*_VIM_, *bla*_KPC_, *bla*_CTX-M-15_, and fluoroquinolone resistance mutations in *gyrA* (S83I) and *parC* (S80I) (24). Kehl et al. reported the persistence of high-risk clone ST147 *K. pneumoniae* in both hospital wastewater and WWTP effluent, indicating its ability to survive treatment processes and enter directly into the environment (33). ST307 is typically linked with *bla*_KPC_, *bla*_OXA-48_, and *bla*_CTX-M-15_, together with fluoroquinolone resistance mediated by chromosomal mutations (25).

Antimicrobial-resistant *K. pneumoniae*-related species strains were not detected in the final treatment stages of the Gavá-Viladecans WWTP, reflecting the efficiency of its secondary membrane bioreactor (MBR) system. Only three antimicrobial-resistant *K. pneumoniae-*related species strains, including ESBLs and carbapenemase producers, were detected in the final stages of the Baix Llobregat WWTP, suggesting a low risk for AMR dissemination through reclaimed water reuse.

The IFAS system is characterized by the coexistence of suspended sludge and attached biofilm, where the prevalence of Gram-negative bacteria is significantly higher than that of Gram-positive bacteria (34). Within these biofilms, high cell density facilitates direct contact and conjugation between microorganisms, promoting HGT (35). These mechanisms may explain the increase in resistant isolates observed at the outlet of the IFAS secondary treatment compared to the primary treatment at the Gavá-Viladecans WWTP, suggesting that the biological reactor may act as a hotspot for resistance enrichment.

Conjugation assays demonstrated that several MDR strains were capable of transferring ARGs to *E. coli*, showing the potential capacity for HGT in wastewater treatment environments.

The strong genotype–phenotype concordance observed for antimicrobial profiles supports the idea that resistance in these strains is mainly driven by well-conserved genetic mechanisms, including specific ARGs. These results highlight the utility of genomic surveillance for predicting AMR profiles.

Additionally, sulfonamide resistance genes were detected in more than half of the strains, consistent with previous studies (36). VFGs were highly prevalent. Nearly 90% of strains simultaneously carried genes associated with iron acquisition (*entB*, encoding enterobactin), fimbrial adhesion (*mrkD*, *mrkC*, *fimC*, and *fimD*), and LPS biosynthesis (*wabG*). These findings are consistent with those of Verburg et al., who reported similar percentages of VFGs and highlighted the pathogenic potential of *K. pneumoniae* in wastewater environments (23).

However, the absence of hypervirulence-associated genes, such as *rmpA* and colibactin, in our isolates is consistent with the findings of Savin et al. (37) and may be explained by the genetic specialization described by Wyres et al. (38). In clinical settings, selective pressure exerted by the host immune system favors the retention of different VFGs, such as *rmpA,* which enhance invasive pathogenicity. In contrast, within WWTPs, these factors represent a high metabolic cost without a compensatory benefit. Therefore, this discrepancy may reflect the ecological adaptation of wastewater-associated populations, in which “classical” lineages predominate. These strains may be better adapted for environmental persistence and opportunistic pathogenicity than for the highly invasive phenotype characteristic of hypervirulent clinical strains.

Furthermore, the absence of significant associations between VFGs and phenotypic traits such as biofilm formation or hypermucoviscosity suggests that these phenotypes are multifactorial and cannot be explained solely by the presence of individual genes. Although fimbrial- and siderophore-associated genes were highly prevalent, their distribution was similar among biofilm- and non-biofilm-forming strains, indicating limited discriminatory value within this collection. Biofilms, detected in 67.6% of the strains, may contribute to environmental survival by providing protection against sub-inhibitory antibiotic concentrations and other stressors commonly present in wastewater systems. Consequently, biofilms may facilitate HGT and co-selection processes, favoring the maintenance and dissemination of ARGs (36). More than half (54%) of the biofilm-forming strains were classified as MDR or XDR. Three genetically similar ST147 strains recovered from the IFAS treatment point across different sampling campaigns were classified as non-biofilm formers under laboratory conditions. Their long-term establishment and persistence within some phases of the wastewater treatment system suggest that factors other than *in vitro* biofilm formation may contribute to environmental persistence. This discrepancy highlights a potential limitation of *in vitro* assays, as environmental conditions such as nutrient limitation, microbial competition, and surface characteristics may induce biofilm formation *in situ* that is not replicated under standard laboratory conditions. Additionally, biofilm assays were performed at 37°C, which is optimal for host-associated growth but does not fully reflect wastewater environments. The use of a 48-hour incubation period enabled the evaluation of more mature biofilm structures, while complementary resazurin assays indicated that shorter incubation periods might underestimate biofilm-associated metabolic activity. Environmental conditions in WWTPs differ substantially from clinical settings, and biofilm dynamics may require longer periods to reach stable and detectable states. This may explain discrepancies between *in vitro* biofilm formation and environmental persistence observed for some strains.

The detection of phylogenetically related STs across different sampling periods, treatment stages, and both WWTPs suggests sustained circulation and resistance to certain water treatments.

The co-occurrence of ARGs with biocide or HMTGs (detected in 36 out of the 37 strains) illustrates the complexity of co-selection mechanisms. Resistance to metals, such as copper, silver, and arsenic, often overlapped with ARG carriage, consistent with previous reports (39), which identified silver-copper resistance as the most common combination. This phenomenon may be related to the continuous exposure of bacteria to a complex mixture of pollutants, including antibiotics, pesticides, phenolic compounds, metals, and chemical preservatives, which can drive cross-resistance (40). In this context, the association between AMR and heavy metal tolerance highlights a critical co-selection dynamic, where 75.7% of the strains carried resistance genes for both stressors, indicating a high potential for environmental persistence (39). This association may be linked to cross-resistance mechanisms, such as reduced membrane permeability and activation of efflux systems. These mechanisms may contribute simultaneously to tolerance against heavy metals such as copper and silver and to resistance to antibiotics, including ciprofloxacin, tetracycline, and chloramphenicol. Consequently, residual heavy metals may act as indirect selective drivers of AMR in wastewater systems.

The *mcr-9.1* gene was detected in a carbapenemase-producing strain that remained phenotypically susceptible to colistin, highlighting the presence of silent reservoirs of last-resort resistance genes. Although not phenotypically expressed under the tested conditions, these genes pose a potential threat, as environmental and clinical selective pressures could trigger their expression through regulatory pathways, such as the *qseBC* two-component system, thereby conferring phenotypic resistance to colistin (41, 42).

Overall, our findings align with those from other European WWTPs, including reports from the Netherlands, Romania, and Spain, where ESBL- and carbapenemase-producing *K. pneumoniae* strains or high-risk clones have been detected in effluents. This suggests that the environmental dissemination of resistant *K. pneumoniae* is a widespread phenomenon (43–45).

Thus, effluents discharged into receiving water bodies could act as vehicles for the spread of ARGs into aquatic ecosystems, reaching agricultural soils through irrigation. This environmental cycle may facilitate the reintroduction of ARBs and ARGs into the food chain, reaching animals and humans.

Our findings emphasize the role of WWTPs as important nodes in the environmental dissemination of AMR within the One Health framework, highlighting the need for integrated surveillance and mitigation strategies. Furthermore, the methodology applied in this study provides a robust conceptual framework that could be extended to include sampling points both upstream and downstream of the WWTP. Characterizing the sources and dissemination dynamics of *K. pneumoniae* across the wastewater continuum is essential for monitoring AMR dissemination.

### Conclusion

Although ARBs and ARGs are not systematically monitored, this study, consistent with previous reports, shows that WWTPs can harbor and may facilitate the exchange of AMR, as suggested by the recovery of MDR and XDR *K. pneumoniae*-related species strains. These include globally distributed high-risk clones carrying ESBLs, carbapenemases, VFGs, and HMTGs.

The frequent co-occurrence of ARGs, HMTGs, integrase genes, biofilm-forming capacity, and conjugative transfer underscores the potential risk of co-selection, HGT, and long-term persistence within wastewater treatment systems.

In addition, the identification of closely related STs across different sampling periods and treatment stages may indicate sustained circulation through WWTP processes.

These results advocate for enhanced genomic surveillance, specifically through routine monitoring of influent and effluent waters for priority markers such as carbapenemases (*blaKPC*, *blaNDM*, and *blaOXA-48*), and the implementation of strengthened One Health strategies to monitor and mitigate AMR dissemination in wastewater environments. To translate these findings into concrete public health actions, the results provide a solid scientific basis for policies aiming to establish mandatory discharge limits for AMR markers and to update safety guidelines for reclaimed water reuse in agriculture and aquifer recharge.

Future longitudinal studies are needed to better understand the persistence and transmission of ARGs and HMTGs across WWTP treatment stages.

## Data Availability

The raw sequencing data are publicly available in the ENA under accession codes PRJEB102516 and ERP183908 (title: Resistant Klebsiella pneumoniae in Barcelona WWTPs). SNPs data are available in Table S1.

## SUPPLEMENTAL MATERIAL

Table S1 spectrum.00621-26-s0001.xlsx SNP differences among strains.

ASM does not own the copyrights to Supplemental Material that may be linked to, or accessed through, an article. The authors have granted ASM a non-exclusive, world-wide license to publish the Supplemental Material files. Please contact the corresponding author directly for reuse.
